# What Happens to All the Bromides?

**Published:** 1897-07-17

**Authors:** 


### WHAT HAPPENS TO ALL THE BROMIDES?

In view of the enormous doses of the various bromides which are administered to some cases of epilepsy and the long period for which they are so often taken, it is a matter of considerable interest to decide what happens to them. Do they accumulate, or are they merely passed through? Dr. Laubenheimer has for a couple of years been making experiments with the object of answering these questions, and for this purpose has examined the urine and the fæces of epileptics both before and during their treatment with sodium bromide with the object of determining how much bromide is retained in the body. It is generally believed that this salt is quickly excreted, but he found that large quantities are retained until the system is saturated with it, which generally takes place in about 14 to 21 days, after which it is excreted nearly as quickly as it is taken. The amount which must be taken before this equilibrium has been established varies with different individuals, and Dr. Laubenheimer believes that the specific anti-epileptic action of the bromides does not show itself until this equilibrium is established—in other words, until the body has received the charge which it is capable of retaining, and that directly excretion exceeds ingestion or this deposit of bromides in the tissue falls below the full charge the fits recur. It is a matter of some importance that the bromides seem to some extent to displace the chlorides in the body. In a patient who was becoming charged with bromide, *i.e.*, was excreting less than he was taking, it was observed that he was at the same time excreting more chlorides than he was receiving in his food. Bromism, then, may not be due altogether to an excess of bromide in the body, but partly to this drug having driven out the chlorides which are so essential to the animal economy—in other words, to chloride starvation.

